# Trust, Risk, and Uncertainty Surrounding the Use of AI in Health Care: Scoping Review of Studies on Public Perception

**DOI:** 10.2196/100747

**Published:** 2026-10-30

**Authors:** Helena Godinho Nascimento, Sultan Abdulkarim, Abel Torres-Espin, Saba Soltani, Geetika Arikati, Samantha B Meyer

**Affiliations:** 1 School of Public Health Sciences, Faculty of Health, University of Waterloo, 200 University Ave W, Waterloo, ON, N2L 3G1, Canada, 1 (519) 888-4567

**Keywords:** artificial intelligence, AI applications, technology, public perception, health literacy, AI literacy, trust, risk-taking, decision-making

## Abstract

**Background:**

While AI has existed for decades, the introduction of generative AI tools and subsequent advancements in their capability and public use resulted in a shift in public awareness and perceptions of AI. In response, the research community has grown increasingly interested in understanding public perceptions of AI use in various contexts, including health care. Public perceptions shape AI acceptance and consequently integration into health care. It is thus crucial to understand public trust in AI and perceptions of risk as we consider its implementation moving forward.

**Objective:**

This study aimed to (1) document the extent of what is known within the literature regarding public perceptions of AI applications in health; (2) interrogate existing definitions and theories of trust being used to guide investigations of public trust in AI; and (3) identify practice and policy considerations and support the design, deployment, and regulation of these technologies in a manner that is trustworthy.

**Methods:**

The Arksey and O’Malley scoping review framework and the PRISMA-ScR (Preferred Reporting Items for Systematic Reviews and Meta-Analyses extension for Scoping Reviews) guidelines were used to review publications between 2022 and 2024 across 4 databases, resulting in the inclusion of 41 papers for review. Key findings were then charted and organized into tables to help answer the proposed research questions.

**Results:**

Public trust in AI in health care was shaped by discourse relating to AI performance and concerns related to depersonalization, loss of human connection, privacy, bias, and transparency. Trust was found to be higher when AI was supporting rather than replacing health care professionals and was influenced by emotional responses, anthropomorphism, demographic factors, and institutional trust. Furthermore, trust extended beyond algorithms to software interfaces, providers, developers, and governing institutions, highlighting the importance of governance and oversight. Conceptualizations of trust varied widely, and trust was defined through vulnerability, competence, benevolence, and integrity. Different theories identified suggested that trust operates across individual, social, institutional, and technological levels. Finally, trust was closely linked to risk and uncertainty, where perceived risk reduced acceptance, while AI literacy, transparency, and explainability mitigated concerns and fostered trust.

**Conclusions:**

Trust in AI is not uniform, and as such, efforts to build and measure trust must account for these nuances to effectively mitigate risk and support informed, ethical AI integration and deployment. On the basis of the findings of this review, we propose a multilevel conceptual framework that provides a general understanding of public trust in AI within a health care context that may guide research and policy and practice considerations. Our findings highlight the importance of considering public trust when implementing AI in health care and may support AI communication strategies, policy development, and trustworthy AI development.

## Introduction

### Background

According to Innovation, Science and Economic Development (ISED), “Artificial intelligence (AI) is a broad field encompassing the science of making computers which behave in a manner that simulates human behavior/ intelligence using data and algorithms” [1]. Furthermore, the Organisation for Economic Co-operation and Development’s Digital Policy Committee defines an AI system as “a machine-based system that, for explicit or implicit objectives, infers, from the input it receives, how to generate outputs such as predictions, content, recommendations, or decisions that can influence physical or virtual environments” [2]. As such, its autonomy as a system can vary, depending on implementation [2]. With AI emerging as a new reality, the potential positive capabilities of AI and AI-based tools have increasingly become talking points among various stakeholders in many industries, including health care. Indeed, the application of AI systems in clinical health care is expansive; for example, there are AI-driven cancer diagnostics, rehabilitation devices, and AI tools used for the detection of heart disease [3]. A recent bibliometric investigation of literature on AI in health care confirms the significance of this field [4]. Applications extend beyond clinical use with generative AI (GenAI) programs, including ChatGPT (OpenAI), Claude (Anthropic PBC), Gemini (Google), and more, providing easy interaction for the public. These AI-based chatbots are being used for the purpose of health data analytics and health information–seeking behavior and to support patients’ health care needs [5]. These examples do not capture the breadth of AI, historically and in the present day, nor the potential of AI. Rather, we introduce them because, as a paper focused on perceptions, we recognize that much public discourse is focused on chatbots and GenAI tools. As such, while this review is inclusive of all that falls under the umbrella of AI, we acknowledge that the very focus of this paper is driven by greater public awareness since the inception of ChatGPT, despite AI as a concept being around for approximately 70 years.

Central to our work, we focus on perceptions of personal health data use in the development of AI applications within health care. Recent data illustrate that students, online panel participants, and certain patient populations care about the way their health data are handled, want to be involved in decisions regarding the use of personal data, and are more interested in the decision-making when they trust the institutions holding their data [6]. Relatedly, these individuals are reported to trust institutions that provide opportunities to “have a say” alongside institutions that are transparent in their practices. Thus, if the public is more trusting of institutions that are transparent in their practices, then institutions that want to implement AI must consider how to build and maintain public trust in a manner that fosters the public’s willingness to share their data (federated and/or centralized) and feed AI applications. Considering public perception in design and deployment is critical. Although determinants and dimensions of trust in AI and automation tend to be similar across domains such as transportation, defense, or general human-robot interaction fields [7-10], research in the health care context is a growing field. This may be because this is a high-stakes context where the application of AI (including generative models) raises unique concerns, risks, and uncertainties, which in turn shape trust for patients, clinicians, and institutions. Accordingly, given the complexity of AI use in health care, we cannot assume that determinants or dimensions surrounding the public’s trust in AI are consistent. This review offers an opportunity to document and synthesize the extent of research in this space, culminating in a potential multilevel framework for future research.

While AI has tremendous potential as a technology for public gain, there are limitations or risks with technologies that may challenge public trust. For example, there has been a propensity of cases in which AI has generated responses that have exhibited racial prejudice or bias [11], leading the public to question the trustworthiness of the technology and claims that AI technologies can inform mechanisms to address health inequities. Furthermore, in the context of health care systems, the sensitivity of data, legal implications of privacy breaches, and consequential risk to public acceptance of future AI applications are critical considerations, especially because public acceptance of AI technologies in the context of health and related willingness to share data are critical if we are to realize the potential of AI for health system improvement (eg, [12]). The development and improvement of AI technologies are dependent on individuals sharing their personal health information, and as such, efforts to handle said data in an ethical and secure manner, fostering trust and AI literacy, should be central to its development and implementation [13]. Addressing and understanding the potential harms (real or perceived) of AI is thus a crucial element in fostering public trust in AI in the interest of population and global health [14].

### Conceptual Framework: Trust in the Context of AI

The definition of trust by Lee and See [15], being the sense that one’s objectives would be aided by an agent when circumstances are precarious, is widely cited across literature relating to trust in AI. However, its application to public trust in AI within a health care context has not been fully explored, particularly because there is vagueness surrounding in whom (or what) the public is placing their trust [16]. Drawing on the definition of trust by Lee and See [15] and other well-established definitions of trust in health literature (eg, [17]), we define trust as the intention of a trustor (entity trusting) to accept risk and vulnerability as it relates to a trustee (entity trusted) based on positive expectations of the trustee, including acting in a manner that is competent (eg, has the skill set to use, interpret, and critically appraise AI outputs); responsible (eg, adheres to AI frameworks); and considerate of the trustee’s best interests [16,17]. We extend the definition to consider trust in individual actors (eg, AI developers) and institutions (eg, those institutions using the technology), both of which can shape how AI is developed and used in a health context [18].

The dimensionality of trust is also considered in our work, whereby trust is a multidimensional construct defined by benevolence, competence, integrity, global trust, relational trust, fairness, communication, dependability, and confidentiality [19]. The dimensions of competence, dependability, and confidentiality are particularly relevant in this work because providers, patients, and other stakeholders are being asked to trust that the AI tool being used to guide clinical decision-making is reliable and accurate. However, dimensions such as beneficence, benevolence, fairness, and integrity—more value-based dimensions—may also be applicable if the individual believes that the AI system is partially or fully conscious or if they consider the individuals and organizations involved in the development of such technologies. These dimensions could also be useful when measuring trust in entities relating to AI, including institutions and organizations implementing, governing, regulating, and developing AI, as well as doctors and health care professionals relying on and using AI. Transparency may also be an important dimension because health care system users and providers are reported to be more trusting if they better understand the AI system and how information is being generated in relation to treatment options [20]. Transparency in this review is conceptualized as the action of being open about the use of AI, how it is or will be implemented; who is involved in the development and use of AI tools, systems, and data collection and storage; and how the AI will process patients’ data, which has implications for better AI governance and ethics.

The literature surrounding trust in AI included in nonhealth contexts identifies various references to the dimensionality of trust and adjacent topics, such as validated dimension sets [7,8], validated scales [21], toolkits [22], and naturalistic models [23]. However, their application in the field of health is limited without broader consideration of why the public trusts technology, or not, and in whom or what they are placing trust. In addition, their focus tends to be inconsistent and based on aspects of the AI itself or the developers, which leaves a gap in other potential drivers of trust in AI (ie, the public institutions promoting its use). We extend the work in nonhealth contexts, focusing on drivers of trust, and consider 5 different types of trust: (1) public trust—trust that a general population holds (eg, the public generally trusting that AI technology within the medical field will perform their duties efficiently) [24]; (2) institutional trust—trust that people have in the actions of institutions around them (eg, trust that the health care system is using AI in the best interest of the communities they serve) [25]; (3) situational trust—trust exercised with certain parties and situation dependent (eg, a person trusting that AI will be of assistance in their own medical surgery) [26]; (4) social trust—trust that an expectation will be fulfilled by the concerned party (eg, a person trusting that AI-powered health chatbots will provide accurate, safe, and helpful medical advice) [27]; and (5) interpersonal trust—trust between 2 people, in which one person has confidence in another (eg, trust in your doctor using the AI) [28]. The distinction in these forms of trust is important in this review to understand what, or in whom, individuals place their trust when trust in AI is established.

Given that perceptions of risk and degrees of uncertainty influence decision-making and impact the acceptance of health-related information [29], the topics of risk and uncertainty are central concepts of focus in this review. As largely nonexperts (in AI or health), individuals are being called upon to trust from a position of vulnerability and uncertainty. Thus, in deciding to trust, there is inherent risk. For example, in considering AI technologies informing cancer diagnosis, risk may include misdiagnoses due to poor data input or interpretation and privacy concerns with regard to user data [30]. Uncertainty regarding AI use in clinical decision-making has already been widely documented, as have questions regarding the extent to which AI is equipped to make the same calls as a doctor would make, especially when it comes to diagnostics [30]. What constitutes a risk will also vary. For example, individuals may be accepting of AI risk of data breaches but aversive to environmental risks of AI as its high energy consumption contributes to climate change. Trust in the regulatory bodies, and uncertainty regarding compliance with legislation, may also influence risk perception. Importantly, we position this review from the perspective that public trust in AI within the health care context may be unique compared to other contexts. For example, risk and uncertainty in a technology may have a direct impact on health and well-being. Therefore, while determinants of trust and measurement techniques may overlap across nonhealth contexts discussed by previous scholars [7,8], documenting trust in AI as it relates to health care specifically is critical to understanding and responding to public perception in the development and deployment of AI in this space.

Finally, and of great relevance to our review, we acknowledge that constructs such as trustworthiness, mistrust, distrust, and confidence are often incorrectly conflated with the construct of trust. Trustworthiness has been conceptualized as preceding trust, where a trustee will evaluate the trustor to be trustworthy (or worthy of trust), and based on that evaluation, chooses to trust or not to trust [31-33]. Mistrust and distrust differ from trust in that they indicate a belief that the trustee will act against the trustor and that the 2 parties will differ from one another [34,35]. Within the health care context, mistrust can be defined as a general apprehension toward the health care system and the belief that the health care system is not acting in a way that aligns with the individuals’ best interests (ie, well-being), while distrust is less general apprehension toward the health care system, often related to negative personal or family experiences with the health care system [35-37]. Moreover, confidence is conceptually different from trust in that confidence may occur when individuals feel as though a system is predictable, regulated, and technically competent, while to trust is to take a risk in the face of uncertainty, unpredictability, and the unknown. In this work, we characterized these concepts as being “adjacent to trust” [38].

### Purpose and Aims

The main aim of this work is to systematically document the literature reporting on public perception and trust in AI within the context of health care. “Public perception” in the context of this review refers to the attitudes, beliefs, and trust as documented in the collected literature that samples members of the public. In 2023, Steerling et al [16] published a review looking at how trust in AI in relation to the implementation in health care had been conceptualized. The authors report that the conceptualization of trust in AI differed between the studies they reviewed, with some focusing on the capability of AI and others on relationships between individuals in cases where AI was being applied. They also note the need for a more holistic understanding of the concept, as it spans different levels and dimensions. We extend the work of Steerling et al, written at a time when only 8 papers were published looking at trust in AI as it relates to health care implementation. We focused on three central objectives: (1) to document the extent of what is known within the literature regarding public perceptions of AI applications in health; (2) to interrogate existing definitions and theories of trust being used to guide investigations of public trust in AI; and (3) to identify practice and policy considerations and to support the design, deployment, and regulation of these technologies in a manner that is trustworthy. In doing so, we document how levels of trust, the dimensionality and types of trust, important concepts behind the decision to trust, and the characteristics of AI are being considered when members of the community purport to trust AI.

## Methods

This scoping review was conducted following the widely used 5-stage framework and methodology of Arksey and O’Malley [39] and the PRISMA-ScR (Preferred Reporting Items for Systematic Reviews and Meta-Analyses extension for Scoping Reviews; Checklist 1) [40]. The PRISMA-ScR provides reporting guidance specific to scoping reviews and is used as a tool to identify key terminology, core concepts, and items relevant to scoping reviews [40].

### Identifying the Research Question (Framework Stage 1)

Data collection was guided by 4 questions (A, B, C, and D; Figure 1). Papers were included if they focused on public trust in AI use, applications, and tools within the health care context. We also included papers focused on concepts adjacent to trust (Multimedia Appendix 1).

**Figure 1.**
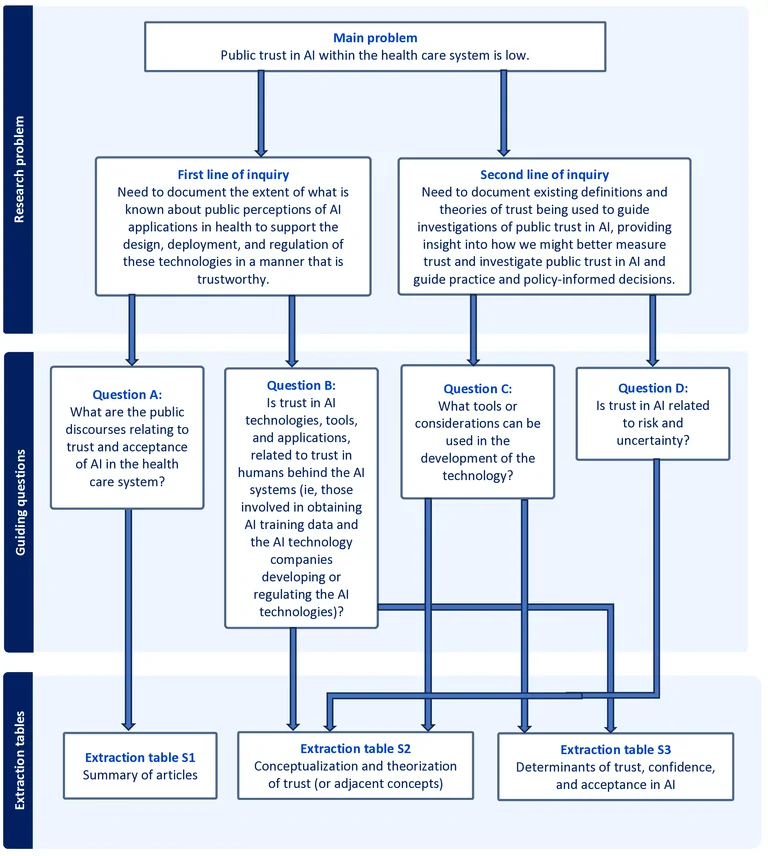
Flowchart of data organization. Organizational map demonstrating how the main problem and lines of inquiry guided the development of the research questions and extraction tables.

### Information Sources (Framework Stage 2)

Papers were collected from 4 databases: PubMed, Scopus, PsycINFO, and Web of Science. All papers were uploaded and screened in Covidence (Veritas Health Innovation Ltd), and duplicates were removed.

### Search and Selection of Sources of Evidence (Framework Stage 3)

Complete search strategies, developed in consultation with a library scientist, can be found in Multimedia Appendix 2. Database selection (subject specific and multidisciplinary) and corresponding search strategies were developed to complement each other, with title, abstract, and subject heading fields used accordingly. Following database searches, each title and abstract was reviewed by 2 independent researchers (HGN and SA). Inclusion and exclusion criteria (Multimedia Appendix 1) were used for the selection of sources of evidence. We limited our search to articles published from 2022 onward as a complement to Steerling et al [16], who covered literature published between 2012 and 2022, and to capture any research developments within the context of the rapid public deployment of GenAI tools in the post-2022 “GenAI era.” The introduction of ChatGPT [41] and the rise in GenAI tools contributed toward the public’s greater access to AI and, relatedly, toward a shift in awareness, public discourse, and policy attention around AI. The fast-moving nature of the field has been acknowledged in previous works [42-44].

Given the lack of conceptual clarity in research on trust and AI and to avoid excluding relevant work on public perceptions of AI, we intentionally included studies that looked at adjacent constructs of trust (ie, confidence, distrust, mistrust, and trustworthiness). Importantly, we acknowledge how these concepts are conceptually distinct from one another (see “Conceptual Framework: Trust in the Context of AI” section). To maintain the integrity of the work, we did not treat confidence (or related constructs) as equivalent to trust. Where studies measured both, we prioritized trust-related findings, while findings primarily derived from the constructs of confidence, mistrust, and distrust were presented as complementary evidence to trust-related findings. Conceptually distinguishing these constructs prevents obscuring what is being measured or studied and unintentionally overgeneralized interpretation of findings. More details can be found in the *Results* section and Multimedia Appendices 3-5.

Finally, prior to title or abstract screening and full-text screening, a mock screening was conducted to ensure consistency while reviewing papers. Reviewers independently screened 20 papers, compared results, and discussed conflicts. The average interrater reliability was calculated using Covidence (title and abstract stage: κ=0.48; full-text κ=0.63).

### Data Charting Process (Framework Stage 4) and Synthesis of Results (Framework Stage 5)

To organize evidence, 3 charting tables were created (Multimedia Appendices 3-5): Table S1 (Multimedia Appendix 3) is a summary of the different studies included; Table S2 (Multimedia Appendix 4) focuses on the conceptualization, theorization, and measurement of trust (or adjacent concepts) as it relates to risk and uncertainty; and Table S3 (Multimedia Appendix 5) reports on determinants of trust, confidence, and acceptance in/of AI. Three reviewers conducted the charting, with the criteria for their assigned extraction sections being discussed in advance. If there were any discrepancies in the extraction, these were reconciled through discussion and explanation, which ultimately resulted in unanimous agreement on what to include (or not) in the extraction.

## Results

In the section below, we present descriptive results and key findings from our charted data to answer the guiding questions. Descriptive results showcase the breadth of existing work and the study characteristics prioritized (Table S1, Multimedia Appendix 3).

### Descriptive Results

#### Selection of Studies

Our search yielded 1288 studies, of which 314 (314/1288, 24%) were duplicates (Figure 2); thus, 974 (974/1288, 76%) studies were included in the title and abstract screening stage. During this stage, 895 (895/974, 92%) studies were deemed irrelevant on the basis of the selection criteria (Multimedia Appendix 1). Accordingly, 79 (79/974, 8%) studies were selected for full-text screening. From the full-text screening, 38 (38/79, 48%) were excluded based on 7 summarized items from the complete exclusion criteria (Multimedia Appendix 1). A total of 41 (41/1288, 3%) papers met the stated inclusion criteria and were included in this review.

**Figure 2.**
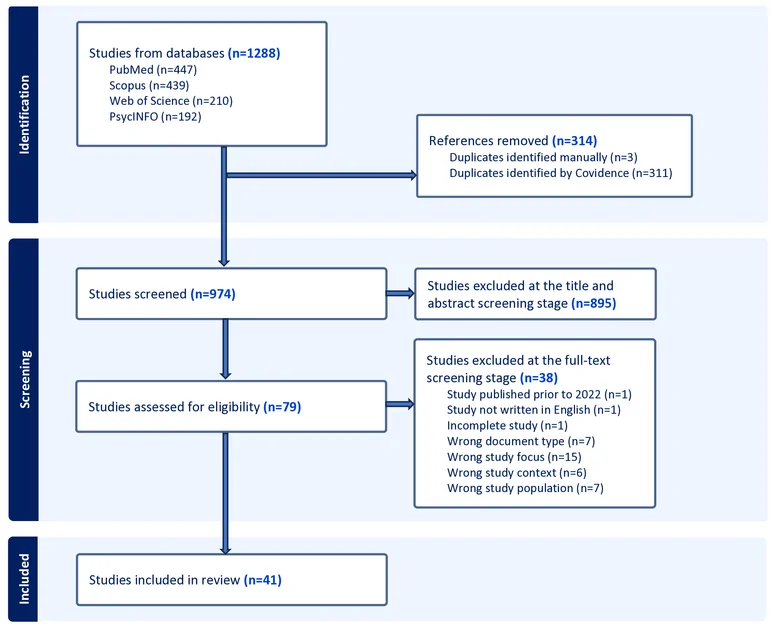
PRISMA-ScR (Preferred Reporting Items for Systematic Reviews and Meta-Analyses extension for Scoping Reviews) flowchart showing the flow of articles from identification (papers collected from databases), through screening (title and abstract; full-text), to inclusion (papers ready for extraction and charting).

#### Study Characteristics

##### Study Design and Focus

Studies were predominantly quantitative (n=33, 80%), with few mixed methods (n=5, 12%) and qualitative (n=3, 7%) studies. Studies largely focused on the concept of trust (n=33, 80%), although the concepts of “distrust” (n=2, 5%), “trustworthiness” (n=3, 7%), “comfort” (n=1, 2%), “mistrust” (n=1, 2%), and “attitudes and opinion” (n=1, 2%) were also identified.

##### Country of Focus (Study)

The following study locations were identified: the United States (n=15, 37%), China (n=8, 20%), Germany (n=3, 7%), Turkey (n=2, 5%), Spain (n=1, 2%), Taiwan (n=1, 2%), the United Kingdom (n=1, 2%), the European Union (n=1, 2%), Finland (n=1, 2%), Italy (n=1, 2%), Estonia (n=1, 2%), Saudi Arabia (n=1, 2%), South Korea (n=1, 2%), Norway (n=1, 2%), Hungary (n=1, 2%), Denmark (n=1, 2%), India (n=1, 2%), and the Netherlands (n=1, 2%). Trust in AI is evidently a globally relevant issue. Importantly, 1 (2%) study (see 1011 in Table S1, Multimedia Appendix 3) was a multisite study that spanned across Finland and Italy; thus, the total location counts exceed the number of studies included in this review.

##### Study Population of Focus

In line with our focus—the public—all papers focused on trust in AI from the perspective of the general population (n=41, 100%). However, some papers did focus on specific groups within the general population, namely students, online panel participants, and specialty-specific patients. This allowed us to identify unique perspectives of diverse populations and various levels of engagement and interactions with AI within the health care system. Specific identities of focus included students (n=7, 17%), patients (n=9, 22%), and parents (n=1, 2%). Additionally, 1 (2%) study included both students and nonstudents (ie, public). This leaves room for future work targeting populations of interest.

##### AI Technologies, Tools, and Applications of Focus

Trust is contextual and, at times, situational [20], and thus, our charting was designed to differentiate between trust in AI technology broadly from trust in a specific AI tool or trust in AI applications. This is important considering that some individuals may trust AI technology generally but not necessarily a specific AI app or tool developed, used, or applied across contexts (Table 1). Furthermore, literature focused on the nuance of trust; that is, whether individuals trust AI tools in one context or application but not others and the rationale behind these attitudes (which may be a limitation of the largely quantitative research designs) is unexplored. In addition, unexplored is how this trust changes over time and the mechanisms of change. Finally, trust in AI may be perceived differently depending on whether a tool is designed for use by health care professionals or directly by patients or other affected members (eg, carers). Although we examined tools for use distinctly by clinicians or patients, differences in tools and contexts prevented direct comparisons of trust indicators across these groups.

**Table 1.** AI technologies and their specific application of focus.

Application group	Specific application
Diagnosis and screening	AI-based Parkinson diagnosis (n=1, 2%), AI-based skin cancer screening (n=1, 2%), AI-based diagnosis for prostate cancer (n=1, 2%), and AI-based diagnosis and treatment (n=2, 5%)
Robotic autonomous applications	AI-based robotic medical implementation (n=2, 5%), AI-based COVID-19 tracing (n=1, 2%), and AI-powered autonomous medical devices (n=1, 2%)
Specialized	AI-based radiology (n=2, 5%), AI-based radiograph or x-ray (n=2, 5%), AI-based cardiotocography (n=1, 2%), and AI-based orthopedics (n=1, 2%)
General	AI technology’s medical applications broadly (n=9, 22%)
Surveillance tracing	AI-powered surveillance in hospitals (n=1, 2%), AI-based COVID-19 tracing (n=1, 2%), and AI-based facial recognition (n=1, 2%)
Information, consultation, and support	AI-based clinical decision support (n=1, 2%), AI-based medical apps (n=3, 7%), AI chatbots for medical use (n=8, 20%), and AI-based health consultation (n=2, 5%)

### Key Findings

Key findings are organized below according to the guiding research questions (Figure 1). Importantly, studies discussed in the guiding research questions contribute to several categories and should not be interpreted as mutually exclusive.

#### Question A: What Are Public Discourses Relating to Trust and Acceptance of AI in the Health Care System?

Many discourses emerged from the literature (Table 2). One major discourse centered on accuracy, reliability, and usefulness (n=10, 24%), where trust was rooted in the belief that AI can make safe and effective health recommendations. A contrasting discourse emphasized depersonalization and the loss of human connection as a key reason for public skepticism (n=6, 15%). Other discourses observed include the role of familiarity and education about AI and how that may shape trust (n=5, 12%), institutional trust in organizations such as the National Health Service (n=4, 10%), and the view of AI as a supportive tool rather than a replacement for human providers (n=3, 7%). Public concerns also included AI efficiency and accessibility (n=5, 12%), and privacy, data security, and bias (n=4, 10%). Emotional reactions to AI’s human-like qualities [45], perceived fairness [46], demographic differences [47], and communication strategies [48] were also reported to shape public perceptions of AI.

Beyond perceptions of the technical abilities of AI, public trust in AI appears to be rooted in beliefs relating to what it means to receive care from another human being [49]. For instance, concepts relating to AI not showing empathy, intuition, or other human-like characteristics led to greater trust in human doctors over their AI counterparts [47,50-53], and greater fears of an “AI takeover,” possibly emerging from broader societal narratives around existential risks [54]. Addressing this concern might involve developing AI with human-like attributes (anthropomorphism, ie, characteristics resembling those of humans [49]). Furthermore, there were inconsistencies in the literature regarding how emotion was perceived. Some studies reported that individuals became more trusting of emotive AI, while participants in other studies did not feel comfortable with machines taking on human roles [46,50].

**Table 2.** Discourses related to trust or distrust of AI.

Category	Summary
Accuracy, reliability, and usefulness of AI (n=10, 24%)	Trust is often based on the belief that AI provides accurate, useful, efficient, or risk-free recommendations; or, conversely, distrust arises when its accuracy or reliability is questioned.
Lack of human touch (n=6, 15%)	Distrust comes from the absence of emotional support, relational care, and personalized attention AI cannot offer, especially compared to human doctors.
Familiarity and education about AI (n=5,12%)	Familiarity with AI, digital literacy, or knowledge of AI increases trust; unfamiliarity reduces it.
Trust in institutions or developers (eg, NHS and technology companies; n=4, 10%)	Institutional trust (or lack thereof) influences how much people trust AI. People may trust AI because they trust the organization promoting it (NHS^a^).
Framing AI as a support tool (vs replacement of humans; n=3, 7%)	People are more likely to trust AI when it is framed as augmenting human care, rather than replacing it.
Efficiency, accessibility, and overcoming human limitations (n=5, 12%)	Trust stems from AI being able to work without fatigue, help underserved populations, offer faster service, or work in dangerous environments (pandemics).
Risk, privacy, and data concerns (n=4, 10%)	Distrust comes from concerns about privacy, algorithmic bias, unknown data handling, or fear of harm from AI decision-making.
Perceived fairness and objectivity of AI (n=2, 5%)	AI is trusted in situations requiring fairness, objectivity, or impartial decisions (avoiding human biases).
Emotional and cognitive reactions to AI’s “human-like” features (n=3, 7%)	People may distrust AI if it seems too sentient, autonomous, or “creepy,” reflecting unease about machines doing human work.
Demographics and inequities in trust (gender, education, ethnicity; n=2, 5%)	Trust varies by group (eg, higher education, male gender, Western origin may be more likely to trust); concerns exist about unequal access and outcomes.
Communication and messaging strategies (n=2, 5%)	Trust is influenced by how AI is introduced; emotionally persuasive or credible messaging can impact public perception.
Media narratives vs public opinion (n=1, 2%)	Media’s positive portrayal of AI does not always match public skepticism, showing a disconnect between media narratives and real attitudes.
Preference for human care despite AI’s capabilities (n=2, 5%)	Even if AI offers advantages (shorter wait times), people still prefer human doctors due to relationships, trust, and loyalty.

a NHS: National Health Service.

#### Question B: Is Trust in AI Technologies, Tools, and Applications Related to Trust in Humans Behind the AI Systems?

The focus of trust is not limited to the AI technology itself but spans algorithms, software interfaces, human actors, and institutions involved in AI development and deployment. Relatedly, we documented literature as it relates to “in what” or “in whom” people are being asked to trust (Table 3). Most studies (n=28, 68%) focused on trust in AI algorithms, particularly in clinical and diagnostic settings. This includes applications such as AI-assisted radiology, cardiotocography [55], symptom checkers [56], mental health apps [57], and autonomous medical decision-making [58]. This suggests that both individuals and researchers care about the accuracy and reliability of AI tools when making decisions and that trust is largely anchored in the ability of AI to replicate or outperform human expertise in specific health care tasks. A smaller subset of studies (n=7, 17%) focused on software interfaces, such as AI chatbots and platforms such as ChatGPT, where trust relates both to the underlying algorithm shaping how AI operates and functions and to how this technology communicates and presents information to its users. Therefore, user experience, clarity, and perceived accuracy of AI-generated content are crucial for trust in these contexts, raising questions about design, transparency, and explainability.

**Table 3.** Focus of trust in AI within health care.

Focus of trust	Key examples from included papers
AI algorithms (n=28, 68%)	AI-based diagnosis [59], AI use in radiology [60], AI-based cardiotocography [55], AI in orthopedics [61], and AI-enabled app for skin cancer screening [62]
Software (n=7, 17%)	AI chatbot [63], ChatGPT AI doctor [64], ChatGPT for health care–related purposes [65], and AI-based medical consultation platforms [50]
Humans involved in AI ecosystem (n=4, 10%)	Trust in dentists using AI in dental radiographic detection [66] and trust in AI PIVAS^a^ vs PIVAS for preparing medications [46]
Organizations (n=3, 7%)	Companies developing or regulating the technologies [51], AI in health care system [45], and acceptance in facial recognition technology [67]
Anthropomorphic AI (n=4, 10%)	Trust in robotic health advisors [47] and humanoid AI robot doctors [57]

a PIVAS: pharmacy intravenous admixture services.

#### Question C: What Tools or Considerations Can Be Used in the Development of the Technology?

Many articles did not report a clear definition of trust, and few relied on theories to guide investigations (Table S4 in Multimedia Appendix 6). Twenty (49%) papers provided a definition of trust, with 10 (24%) focused on trust in ChatGPT or chatbots. Seventeen (41%) papers defined trust in relation to technology (eg, ChatGPT), generalized trust, specialized trust (eg, experiential trust), or defined concepts adjacent to trust (eg, trustworthiness). “Generalized trust” reflects definitions of unspecified subjects of trust (eg, trust is about believing that another entity will commit to what is expected of them). Within Table S4 (Multimedia Appendix 6), we reflect on the chosen language, words used to define trust, and related concepts, as this information may help inform future trust strategies. For instance, how we respond to challenges of trust will vary if trust is considered an action or a response, if trust is the responsibility of the trustee or the trustor or trusted entity, and if trustworthiness is a characteristic that can be expected of an AI machine.

Thirty-one (76%) studies did not identify dimensions of trust. Of the 10 (24%) papers denoting dimensions, trust was discussed in relation to 1 key domain: trust in technology itself (eg, ChatGPT for health care; ). Dimensions of trust are consistent with the broader trust literature [19] and include dependability (2/10, 20%), confidence (1/10, 10%), confidentiality (2/10, 20%), fidelity (1/10, 10%), competence (5/10, 50%), honesty (1/10, 10%), compassion (1/10, 10%), privacy (1/10, 10%), reliability (1/10, 10%), communication (1/10, 10%), benevolence (3/10, 30%), integrity (3/10, 30%), and ability (2/10, 20%). Additionally, factors such as transparency, fairness (to avoid perpetuating health inequities and discrimination), and health privacy (health data protection assurances) emerged as essential considerations for the development and implementation of any AI tool or system within the health care system [20,68-70]. AI explainability has been investigated with regard to technical methods for improvement; for instance, explainable AI has been studied alongside specific reasoning methods to better develop deep learning models that conduct medical diagnoses [71].

Twenty (49%) studies included theories that informed their conceptualizations of trust (Table 4) that can be categorized into cognitive, social, organizational, and technology-related. This is because some theories, such as the technology acceptance model, were distinctly technology oriented, while others, such as social comparison, lend themselves to more social contexts. Privacy calculus theory and the knowledge deficit model, for instance, involve thinking patterns and processes, thus being more cognitive rather than social. The theory of organization-public relationships and delegation theory may be parallel to one another: the dissemination of a piece of information to other people, implying something more organizational than simply social.

**Table 4.** Different trust-related theories grouped into cognitive, social, organizational, and technology-related categories.

Theory group	Theories
Cognitive theories	Privacy calculus theory [50,72], status quo bias perspective [73], knowledge deficit model [73], and elaboration likelihood model [74]
Social theories	Algorithm aversion theory [46,52], diffusion of innovations theory [75], self-categorization theory [67], social comparison [76], attachment theory [45], uncertainty reduction theory [74], folk theory [74], social response theory [77], theory of acceptable risk [78], and Siegrist’s causal model of public acceptance [78]
Organizational theories	The theory of organization-public relationships [79] and delegation theory [80]
Technology-related theories	Technology acceptance model [24,48,49,62,67,79,80], trust in automated systems theory [81], framework for human-related factors, AI-related factors, and contextual factors [56], human-machine communication theory [75], Unified Theory of Acceptance and Use of Technology model [45], trust in technology model [77], perfect automation schema [82], and imperfect automation schema [82]

#### Question D: Is Trust in AI Related to Risk and Uncertainty?

Understanding the way risk and uncertainty work in conjunction with trust can help in the creation of trust strategies and ensure new AI technologies can mitigate, rather than increase, public risk through appropriate safeguards. Twenty-four (59%) studies included information on trust as it relates to risk and uncertainty, leading to 3 key themes outlined in Table 5.

Individuals appear to be more accepting of AI in less invasive specialties such as dermatology or radiology than in specialties such as surgery where the consequences of error feel more severe [47]. Health anxiety may also impact public trust in AI (eg, high levels of anxiety resulted in lower acceptability of AI-generated diagnoses [76]). However, findings suggest that under mortality salience, such as during the COVID-19 pandemic, trust in AI seemed to increase, which may suggest that during crises, some people prioritize rational efficiency over human-like connection and reassurance [83]. Privacy concerns also play an important role, with some individuals choosing to trust AI over a human doctor when it came to sharing sensitive data, stemming from beliefs that there is a lower risk that sensitive data would be misused or handled carelessly [50]. These concerns relate to perceptions of risk, a concept that is reported to influence trust in AI and related AI use [84]. Trust in AI may also be in part shaped by inherent characteristics of individuals (ie, personality traits), such as being “risk prone” and “risk averse,” as speculated by a recent cross-sectional study [49]. Furthermore, evidence suggests that gender may shape public perceptions of AI, and, consequently, trust in AI (eg, highly educated men may be more trusting of AI compared to women [47]; women, younger patients, and less-educated patients may be less comfortable with AI-based care [61]; men may be more trusting of AI doctors, whereas women may prefer human doctors [75]; women may have less positive attitudes toward autonomous robot surgeons than men despite human likeness [83]; and men may possess a higher baseline knowledge of AI, facilitating increased trust and reduced distrust in AI [85]). Relatedly, research shows that higher understanding of AI, informed consent, AI literacy, and prior AI experience may result in higher trust in AI [46,85]. Perceptions of expertise in the subject area may also impact trust. For example, 1 (2%) study compared AI, a human expert, and a human novice and found that trust in the human expert was higher than in AI, but trust in AI was higher than in the human novice [82].

AI literacy (ie, understanding of AI application and limitations) and level of digitalization across countries may shape risk perception and are thus key to understanding public perceptions of AI in the health care system globally. Indeed, individuals’ expectations of AI systems, tools, and technologies may vary across different levels of knowledge and understanding. Accordingly, individuals who believe AI systems are fully autonomous, conscious, and independent may expect that the system processes some levels of moral compass, while those who believe these systems are purely operationalized bias algorithms may not have the same expectations. Also important in shaping trust in AI via AI literacy is the role of AI in producing, amplifying, and combating infodemics (including misinformation and disinformation online).

Finally, AI legislation, which varies across jurisdictions and is evolving with technology, should be considered in relation to risk and uncertainty. Some studies allude to the potential impact of inconsistency in legislation on public perceptions of AI and, consequently, their trust and acceptance of AI. For example, 2 studies included in this review, 1 (2%) from Germany [45] and 1 (2%) from Denmark [85], acknowledged the unique context of their respective countries in shaping the interpretation of their results, particularly regarding the generalizability of the findings.

**Table 5.** Common themes on trust in AI with specific examples.

Themes	Examples
Concepts relevant to the relationship between trust in AI and risk and uncertainty	Education level, gender, age, ease of use, accuracy, reliability, misinterpretation and overreliance, privacy risk, familiarity with AI, ethical considerations, informed consent, independent AI, concerns about AI replicating human decision-making, information control, competence of person behind AI, objectivity, uncertainty about health status due to online health consultations, lack of transparency, lack of explainability, lack of regulation, earliness of technology, misuse concerns, accountability concerns, and mortality salience [48,51,59-61,63,66,67,72,73,77,78,80,81,83,86,87].
Explanations for how trust and risk are related	Risk perception having an increase can lead to less AI use and vice versa [84]; higher understanding of AI leads to higher trust [48]; people tended to focus more on AI risks than benefits despite knowledge of AI, trust related more to risk than benefit perception [73]; perceived risk has a negative effect on trust [72]; users are uncertain about interacting with AI [74]; low- vs high-risk context affects risk perception [51]; and trust in information sources can be the difference between someone being willing or unwilling to act on information, and this carries over to AI [57].
Potential risk perceptions	Misinterpretation and overreliance [65] and medical AI can lead to less invasive surgery and therefore less risk for patients [52].

## Discussion

### Principal Findings

The objectives of this review were (1) to document the extent of what is known within the literature regarding public perceptions of AI applications in health; (2) to interrogate existing definitions and theories of trust being used to guide investigations of public trust in AI; and (3) to identify practice and policy considerations and to support the design, deployment, and regulation of these technologies in a manner that is trustworthy.

Recent public discourse relating to trust and acceptance of AI in health care suggests that trust in AI is multifaceted, shaped by both practical expectations and deeper emotional and relational considerations. Discourse suggests that the public is generally hesitant toward technology, with anthropomorphism emerging as an overarching theme. Accordingly, AI may be more trusted if introduced as a support system rather than a replacement for human health care professionals [55,67]. The development of guidelines that oppose the overreliance on AI by health care professionals (and noting parameters for use) may be a mechanism for fostering public trust in AI technologies in health care [55]. This should be considered during provider training on AI tools (automation and diagnostic), dialogue regarding how much human involvement is needed in AI applications, and considerations of liability and fault in the case of negative outcomes.

Public trust was found to vary across trustors and on the basis of who is being trusted, whether the object of trust is a tool, something a doctor uses in practice, or the institution considered to be behind the technology. Some studies note that trust in AI depends on institutional trust; this could include trust in AI developers within AI technology companies and in any regulatory body in place to ensure AI is deployed ethically [51,55]. Low trust in these institutions may lead to decreased AI trust or, alternatively, trust in AI over distrusted (or less experienced) human health care professionals [62]. Findings from our review support the distinction between algorithms and the people and systems behind them as related to trust, especially if we consider accountability for the outcome (good or bad) in the use of such technologies and where responsibility lies. This finding aligns with prior work where a decrease (or complete loss) in trust in one part of an automated or artificially intelligent system can lead to less trust in other aspects of the AI or surrounding the AI [88]. These dynamics of trust demonstrate that trust may not necessarily be relegated solely to the AI system [89]. Centering the human in conversations of AI development and application, which can be done through understanding the different aspects of AI that people look to trust and the perceived risk they experience with AI use, aligns with approaches in software and human-centered design and may be used to inform future efforts in upholding that trust, thus supporting human autonomy and decision-making [90,91].

In line with the review by Steerling et al [16], inconsistencies in and absences of trust definitions were noted. Further aligned with the review by Steerling et al [16] and other works [7,8], reviewed studies focused on different trust dimensions and contexts or aspects of AI upon which trust is placed. Given the complexity and multidimensionality of trust and how it may be shaped by individual, organizational, and cultural contexts [15], we recommend that studies investigating public trust in AI within the health care context clearly define and conceptualize or operationalize trust within their work. This will foster comparability across studies, assist in the development of measures of trust, and provide context with which to interpret study results. Furthermore, we note that across definitions and trusted entities, trust is shaped by the individual trusting (trustor) and the situation or condition of trust. Often, these conditions are those where there are risks, uncertainty, and complexity involved [45,65,72,77,80,92]. This aligns with previous work investigating trust in automation [15], where trust is conceptualized as guiding behavior amid uncertainty, complexity, and change (ie, trust can act as a guide to deciding to accept a tool or technology that is AI-based or AI-driven for health care). Moreover, presented definitions of trust also focused on characteristics of the AI tool, technology, or application (eg, competency or reliability of the tool), or those using the AI tool or technology (eg, competence and/or reliability of the health care system or the health care providers within the system). Overall, the literature suggests no clear operational definition of trust in AI; and while it is unlikely that a single definition of trust in AI will ever materialize, it is critical that researchers provide a conceptual framework that guides their work—that is, the dimensions of trust in focus, the trustee, the context, and relevant concepts. These factors are critical to both understanding and responding to challenges of trust across a complex landscape with many actors, applications, populations, and contexts.

Despite the review by Steerling et al [16] highlighting the need for a more holistic understanding of trust relating to its different levels and dimensions, and these being key considerations when measuring or assessing public trust, most of the literature reviewed did not take note of these dimensions, with most of the studies we collected showing no clear dimensional framework being applied. This should be a priority inclusion for future studies. A starting point may be parsing out value-based versus non–value-based dimensions. For instance, we propose that dimensions of trust can be classified as both value-based (ie, honesty and compassion) and non–value-based dimensions (ie, confidence, privacy, reliability, and ability), with the understanding that value-based dimensions are more applicable toward someone or something that may be partially or fully conscious. It is also interesting to note that some dimensions may be more difficult to categorize as either value-based or non–value-based. For instance, fidelity can be interpreted as factual accuracy or other measures of correctness by focusing on whether technology is (or is not) faithful to reality (eg, is a magnetic resonance imaging machine producing images with high fidelity?)—a non–value-based dimension. On the other hand, fidelity may also be interpreted as loyalty, in which there is an assumption that AI may have some level of consciousness upon which we can expect loyalty—a value-based dimension. Not clearly defining or conceptualizing trust or adjacent concepts to trust can also further contribute to the incorrect conflation of these different constructs and impact the interpretation and application of findings within the field of AI within health care. Since conducting our literature search, new work has investigated how trust in AI-powered medicine can be placed in various contexts, such as emotive aspects of the AI and the structure or cognitive capacity of the AI in ways that diverge rather than being joined [93]. This illustrates the importance of distinguishing between value-based and non–value-based dimensions of trust.

We also note the vast interdisciplinary interest in using theory to explain individuals’ perceptions and behaviors as they relate to AI use and acceptance within the health care system. Across the 20 (49%) papers incorporating theory, there is a notable overlap in theories and frameworks applied. While many theories do not explicitly acknowledge trust as a central factor (eg, trust is not evident in the title of the theory or framework), trust emerges as a key component within the theory. This finding presented an opportunity to generate a multilevel framework that integrates relevant aspects of the collective theories (Table 4) and can advance investigations and related understandings of trust in AI (Figure 3). In Figure 3, we portray a sociotechnical framework for looking at trust, whereby trust is informed by the interactions between people, technology, and processes across all levels rather than AI technology alone. This aligns with earlier discussion in the literature surrounding trust in AI [94,95], pointing to a system-level dynamic [96] rather than one that is only between the user of the AI and the AI itself. Our framework identifies 4 different levels through which trust in AI may be better understood and at which level-specific interventions may be implemented. Level 1 is the individual and cognitive level that includes theories that speak to individual perceptions, cognitive biases, and thinking mechanisms that may shape how individuals evaluate risks and uncertainty and ultimately choose to trust AI (may focus on specialized trust forms such as cognitive and emotional trust). Level 2 is the social or interpersonal level, which includes theories that emphasize the role and importance of social dynamics, group identities, and emotional bonds and how these influence public perception and trust of AI (may focus on the trust individuals have toward AI doctors as well as human doctors relying on AI for their practice). Level 3 is the organization or institutional level that includes theories to help explain how trust is built or eroded through structural factors such as AI policies or relationships with institutions, organizations, or companies developing, deploying, or regulating AI systems (may focus on institutional trust and look at public perceptions of health care organizations and technology companies). Finally, Level 4 is the technological or system level that includes theories to examine the role of specific attributes or characteristics of AI systems, such as reliability, transparency, explainability, and controllability, that may shape trust levels (focus on trust as it relates to the AI system properties and thus trust forms that focus on the performance and ability of technology or machines to meet expectations). A multilevel theoretical framework for trust in AI can help highlight areas for interventions at varying points of vulnerability and uncertainty, becoming a crucial tool for fostering public trust in AI.

Figure 4 acts as a conceptual model and hypothesis generator for future research. It suggests that the technological and system level directly influences the individual and cognitive level, while the latter level interacts dynamically with the social and interpersonal level. The dashed arrow reflects how institutional policies may affect the interaction between individuals and public trust in AI in health care. We note that the moderation and causation claims illustrated in Figure 4 were not empirically evaluated in the reviewed articles, nor assessed by our team in preparing this review; instead, these are presented as hypothesized relationships that warrant testing in future research.

**Figure 3.**
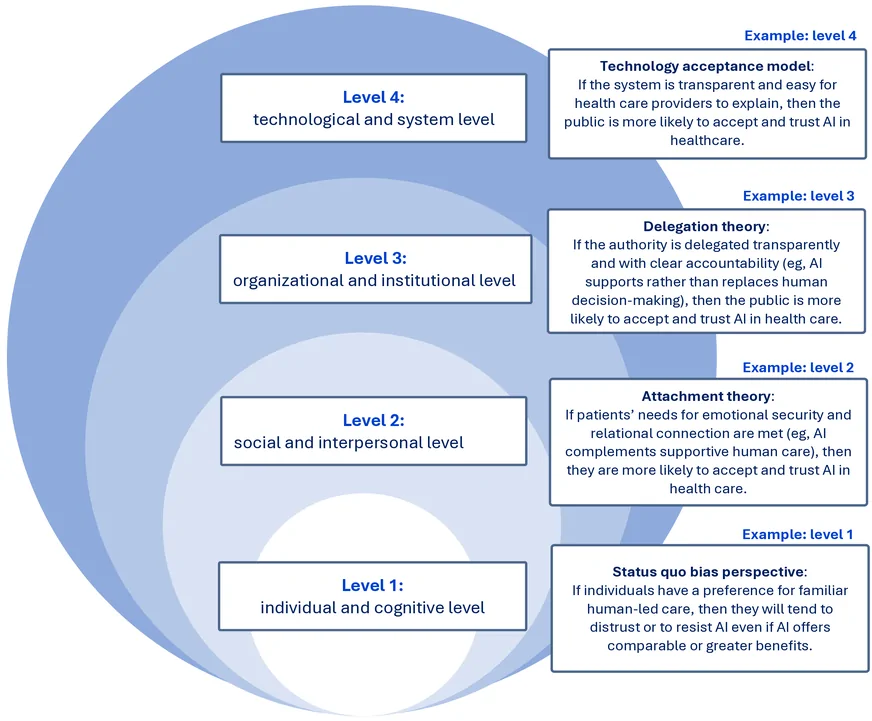
Proposed multilevel framework.

**Figure 4.**
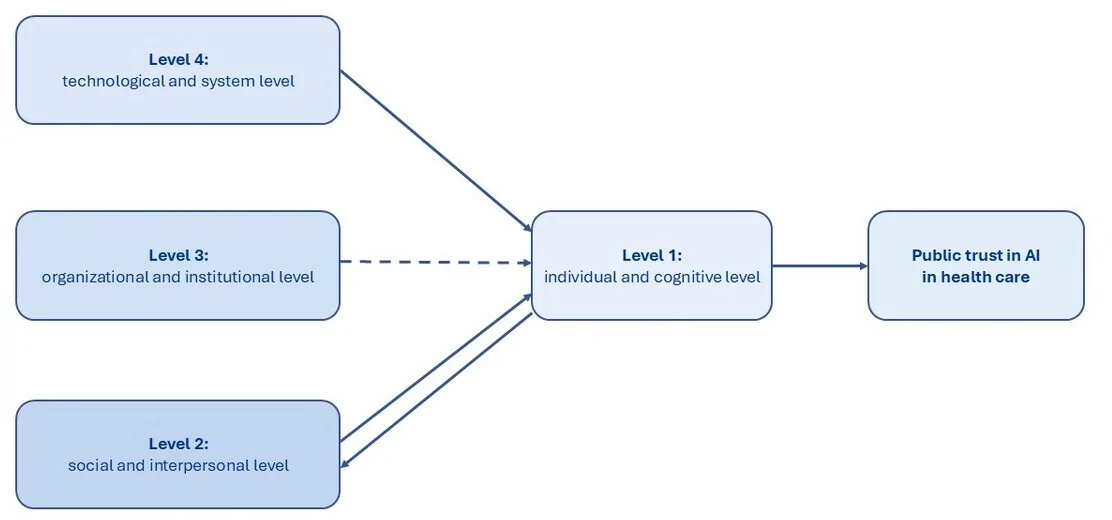
Conceptual model and hypothesis generator for future research investigating trust in AI. Solid arrows indicate dynamic interactions and causal directions; the dashed arrow indicates that institutional policies moderate this interaction.

Literature relating to risk and uncertainty within the context of AI use within health care is also of great relevance. Public trust in AI is linked to perceptions of risk and uncertainty, shaped by context, individual differences, and the specific medical scenarios involved. Some other work has investigated individual differences as they relate to trust levels [97]. For example, recent work has found that personality traits such as propensity to trust and risk aversion are important characteristics in shaping trust [98]. Future work should investigate individual characteristics shaping trust within the context of AI in health care. Factors relating to public trust in AI also included AI literacy and AI explainability. These findings align with research arguing that AI is perceived as a “black box” and in technological infancy, both of which make it challenging for patients to adequately understand how AI works, consequently affecting trust [52]. Opaque AI (ie, “black box”) systems may create uncertainty regarding who bears responsibility for a clinical decision and may impact public perceptions of risk and trust. Responsibility may lie with clinicians, the institutions in which they operate, the developers of the AI itself, or among other stakeholders who were involved in the development, deployment, and regulation of AI systems. Ensuring transparent communication about accountability may aid in addressing ethical and safety concerns regarding AI application in health care [99].

AI literacy is likely to vary across roles in health care. For instance, radiologists using AI-supported imaging may require knowledge and skills that allow them to identify when a model holds bias, and to identify and resolve discrepancies in an AI system’s decisions [100]. Thus, as AI develops, training and education on AI is critical for all health care providers. Supporting health care professionals through professional development and trainees as part of ongoing curricula development will be critical. This will involve identifying the skill sets and knowledge required for different fields of medicine. For example, training a general practitioner on AI-based tools for documenting patient histories will vary significantly from training an ophthalmologist who might need it for interpreting AI image reports. As AI advances, training may need to be tailored to the specifics required within unique professional roles. Patients’ trust in AI systems may depend on the medical professionals applying the tools, underscoring how specific roles will require specified training to foster public trust. Future research should examine these concerns.

We also note that uncertainty related to public trust in AI may be shaped by changes in patient-doctor relationship dynamics, possibly associated with the transition of patients from passive roles toward more active roles within the health care system (eg, questioning of authority, advocating for their own health, patient-generated health data), facilitated to some extent by greater access to health information [101,102]. This is exacerbated by increased the spread of misinformation online [103] and declining institutional trust following the COVID-19 pandemic [104]. AI as a tool for (re)building trust in health care (eg, by replacing health officials and organizations where trust is lost) is an area of investigation that might be explored in future research. For example, criticism from women about limited resources targeted toward women’s health issues has led to a growth in Femtech, that is, technology, including AI software, that addresses health and wellness concerns that solely, disproportionately, or differently affect women, girls, nonbinary folks, trans people, and those assigned female at birth.

Other factors relate to risk and uncertainty, notably health literacy, AI literacy, and level of digitalization across countries (ie, understanding of AI application and limitations), which shape public perceptions of AI in the health care system globally. This is supported by efforts from global organizations such as the World Health Organization (WHO), which is currently relying on AI-supported tools to identify and assess narratives and “information voids,” and the current status of misinformation and disinformation, respectively, which affects how people receive information needed to make healthy choices [105]. Contrarily, evidence also suggests that large language models (eg, ChatGPT) may generate misinformation [106], which leads to confusion among the public regarding the role of AI in health care [105].

We also noted the potential relationship between loss of trust in the health care system and reduced health and AI literacy. For instance, it is possible that lost trust in health care (and other social institutions), driven by a lack of understanding or knowledge of how these systems operate, its extent, and limitations, might result in greater distrust of AI applied to health, since tools or technology introduced within the health care system may be perceived as an extension of the system itself. While one study included in this review ([62]) suggested that introducing AI technologies within the health care system may be a solution to individuals’ lack of trust in human health care professionals, it is still unclear whether proposed technology by public health authorities will be accepted by individuals who are not trusting of those same authorities. Furthermore, more work is needed to investigate literacy and, specifically, how education and training within countries might influence public acceptance of technologies (eg, considering how the University of Helsinki nationwide course on AI literacy has impacted trust at a population level). Future studies might investigate how health and AI literacy, as well as institutional trust, shape trust in AI. Our results highlight the importance of understanding countries’ differences in their levels of digitalization, as well as in their legal and ethical approaches to AI, which may help establish a culture that supports ethical governance of AI use, application, and development, thus reducing public uncertainty.

Finally, health care system structure, which is country specific, and the role it may play in shaping perceptions of trust is important to consider. For example, in countries where the health care system is not accessible to all, AI may create unique opportunities for increasing access to care (eg, by identifying system efficiencies). In this scenario, increased access to—or availability of—health care services because of AI may increase trust in AI [47]. Alternatively, in areas where AI might not afford obvious benefits, citizens’ skepticism of AI and how it may impact their current care may increase, as was the case in the Netherlands [47]. Access to health care and how it may shape AI acceptance and trust was not directly captured from our review. However, it highlights the potential role that AI legislation, digitalization levels, and health care structure may play in shaping trust. Future work should investigate these relationships and associations further.

### Implications for Practice and Policy

Our review provides a synthesis of the various factors that influence trust, or lack thereof, in AI technologies in patient care in a manner that might guide policy and practice communities around AI integration in the health context. We illustrate that the integration of AI tools into care needs to be accompanied by transparent communication regarding how AI will be used. For example, to address concerns about privacy and confidentiality, practitioners should find mechanisms to disclose how data will be used and stored and obtain written consent prior to use. For example, the use of a tool (eg, AI scribe) might be used in the patient’s presence, with the practitioner explaining the reasoning for each question or prompt asked. It is also important for patients to know that they can opt out of the use of AI during their treatment process. Furthermore, concerns about a lack of human-like characteristics (ie, emotional support or personalized attention) might inform how a provider introduces AI—as a tool to gather evidence and inform tailored treatment but not as a replacement for provider engagement in care. Rather, these tools might free up time for greater face-to-face engagement in clinical practice.

Health care providers may also gain public trust by promoting AI literacy and awareness of current AI guidelines within their country and clinical practice that are transparent, mitigate risk, and lead to patient benefit. These strategies may support ethical, informed, and consistent health care delivery practices, wherein AI is responsibly integrated and implemented (eg, providing transparent information about the human oversight and continuous monitoring of AI procedures to mitigate risks [107]). Explaining the benefits of using AI, being transparent about risks, implementing AI health literacy training, and ensuring patients feel actively involved in their treatment process are all effective strategies for promoting trust in AI use in clinical settings. Protecting patients’ sense of control and autonomy in their care plan may reduce uncertainty and may increase trust in AI-integrated health care.

Finally, researchers and policymakers should operationalize AI literacy as a set of measurable and role-specific competencies as opposed to a general awareness of AI. More specifically, we recommend that these competencies include understanding how AI systems work (ie, what they can do and not do), how to critically evaluate AI outputs, how to protect privacy of users, how to recognize bias in the algorithms, and when human oversight or intervention is needed, although the extent of training and competency will vary depending on the role of the individual in clinical practice [108-110]. We propose that AI literacy be integrated into training (eg, medical school) and as part of ongoing training (eg, professional development) and its effectiveness evaluated through practical tasks and validated measures that account for social groups and role differences [108-110].

### Gaps and Areas for Future Research

Despite evidence suggesting that AI training data are biased and thus AI may perpetuate harm against racialized and other marginalized populations, the papers captured in our study did not investigate these harms as they relate to trust, and this is an area for future investigation. Culture as a determinant of trust is also an area of exploration; for example, some literature suggests that trust in AI levels was higher among Dutch or Western participants compared to those with non-Western immigrant backgrounds [47], aligning with the argument that culture impacts trust levels [84], but this remains underexplored. More work is also needed to directly investigate how trust in AI varies by gender and other sociodemographic characteristics, including race, ethnicity, or intersecting social identities, as it is imperative that future work investigates the role of AI in perpetuating unfair treatment, discriminatory practices, and how this shapes the trust of marginalized groups.

Few papers investigated public trust in individuals behind AI (eg, developers and engineers), as well as governing bodies regulating AI, leaving room for research in this area. Moreover, despite papers reporting on the public’s lack of trust, distrust, and hesitancy toward the use and overreliance on AI in the health care system, very few studies provide recommendations or solutions to foster or develop public trust in AI. Furthermore, only 1 study included in the review investigated strategies to increase AI technology adoption [86].

Finally, few studies included in our review used social media to recruit participants, and none used social media scraping as a method or tool for data access. This may be the result of challenges with accessing data (ie, closure of APIs and severe restrictions on data access across platforms), which limits the use of social media posts as data and researchers’ ease in scraping social media to study human and social phenomena [111]. Given that social media currently plays an important role in capturing the more unfiltered and honest opinions and thoughts of individuals, future researchers are still encouraged to design a study that collects opinions, thoughts, and anecdotes directly from social media platforms (eg, Reddit) to further explore public perception of AI use in health care as it relates to risk and uncertainty, which can be used to complement the peer-reviewed evidence we identify in our review.

### Limitations

We did not register a protocol in advance of this review. To maintain the integrity of the work, detailed reports were kept on the development of the review and decisions made along the way that all authors could refer to at all stages of the review. Consistent with our methodology, we did not conduct critical appraisal of the sources of evidence. As such, we make no claims about the quality, strength, or rigor of each of the studies included in this review. We present information extracted from the included studies on reported limitations and methods used, including how variables were measured, that readers may choose to review (Table S1, Multimedia Appendix 3). While search terms were selected to create a comprehensive and exhaustive list of relevant studies for screening and later extraction, every systematic search strategy has limitations and accommodations in its development; therefore, it is possible that some relevant studies may not have been captured by our search. To minimize this risk, we consulted with a library scientist to help in the development of search strategies. While gray literature (ie, government documents, reports, and books) is a valuable source of information, especially within the context of public perception and public opinion, gray literature sources were not included given the scope of the work. Future works may choose to complement this review by scoping current gray literature on public perceptions of AI use in health care. The literature collected in this review is typically based on patient and student populations, along with data from online panels. These do not necessarily account for direct representations of the “general public,” as people can be from varying backgrounds and ages that would not necessarily be student populations, nor would they necessarily be polled in a clinical setting. Future research with representative sampling is required to confirm whether the findings here have great generalizability. The methodological choice to narrow the scope of the review to 2022 to 2024 publications and literature focusing on the health care context may exclude important non–health care and pre-2022 works. Future reviews may choose to look at these to see how public trust in AI may differ (or not) across other contexts. We recognize that the title or abstract interreliability score of κ=0.48 is weak. Ongoing discussions about how AI is defined and used in health care, as we navigated the complex and evolving nature of trust in AI, led to refinements in the inclusion and exclusion criteria during screening. This increased interrater disagreements and explains the observed κ statistic. To resolve screening conflicts, a third reviewer was introduced, and group discussions were held to reach a final consensus.

### Conclusions

This review systematically examined the literature on public trust in AI within the health care context, highlighting how trust is defined and theorized, what drives its formation or decline, and the subject of trust under study (in whom, or in what, people are being asked to trust). It also explored how risk, uncertainty, and public discourse influence public perceptions of AI in health care. Our review provides evidence of the need to consider public trust when governments, policymakers, and health care providers are making decisions about how and when to implement AI within health care contexts. In addition, we provide a potential multilevel conceptual framework that synthesizes the theories surrounding trust found in this review; this is intended as a tool for researchers and practitioners to target interventions along with creating an understanding of trust in AI.

While there is risk in the deployment of AI-based systems in health care, and understanding this risk is paramount in the way AI is handled moving forward, there are still many benefits that AI can bring to the health care space, and asking questions regarding trust, uncertainty, and risk only serves to improve the way in which we handle AI rather than discard it. Our findings support that trust cannot simply be assessed by asking whether people trust AI or not and that there are different scenarios where trust in AI varies. Public trust in AI is contextual, influenced by the characteristics of individuals, characteristics of the AI tool, and characteristics of its application. As such, research and design efforts aimed at building trust must carefully account for these nuances, grounding their work in the populations and contexts under study to ensure trust is better understood, and risk and uncertainty mitigated. Finally, our review works as an update on trust in AI within health care literature in the “GenAI era,” which centers public perception of AI, following previous works (eg, the review by Steerling et al [16]), and it is not intended as an exhaustive review of all trust in AI literature. Given the pace at which research is being produced in this space, it will be important to regularly update the literature as technologies evolve.

## Supplementary material

10.2196/100747 Multimedia Appendix 1 Complete inclusion and exclusion criteria.

10.2196/100747 Multimedia Appendix 2 Developed search strategies.

10.2196/100747 Multimedia Appendix 3 Summary of papers.

10.2196/100747 Multimedia Appendix 4 Conceptualization and theorization of trust (or adjacent concepts).

10.2196/100747 Multimedia Appendix 5 Determinants of trust, confidence, and acceptance in and of AI.

10.2196/100747 Multimedia Appendix 6 Trust definitions based on identified themes.

10.2196/100747 Checklist 1 PRISMA-ScR checklist.

## Abbreviations

GenAI: generative AI
ISED: Innovation, Science, and Economic Development
PRISMA-ScR: Preferred Reporting Items for Systematic Reviews and Meta-Analyses extension for Scoping Reviews
WHO: World Health Organization
