# Supplementary material for: Trust, Risk, and Uncertainty Surrounding the Use of AI in Health Care: Scoping Review of Studies on Public Perception
Source: JMIR AI. 2026 Oct 30;5:e100747. doi: 10.2196/100747 (PMC13721850; doi:10.2196/100747)
Supplement: Supplementary file 2 [file ai-v5-e100747-s001.docx]

# Trust, Risk, and Uncertainty Surrounding the Use of Artificial Intelligence in Healthcare: a Scoping Review of Studies on Public Perception

## Appendix 1. Complete Inclusion and Exclusion Criteria

### A1.1 Inclusion Criteria:

General

- Include empirical research papers that are peer-reviewed.
- Include papers in English only.
- Include papers published between 2022 and October 2024 (inclusive of 2022).

Population

- Papers that focus on public trust (ie, general population) including trust from patients within the healthcare system and other users of the AI technology.

Context

- Papers focusing on the context of Healthcare.

Focus

- Papers focusing on trust in AI technology, tools, applications, and or trust in individuals/institutions behind the AI technology/ tool.
- Papers that look at the nature and extent of Trust in AI
- Include papers that focus on concepts adjacent to trust (e.g. papers on confidence, distrust and mistrust in AI; papers on trustworthiness of AI).
- Include papers that measure trust in AI (including autonomous robots)

Outcome

- Papers that look at health implications as they relate to trust broadly (ie, papers that look at social and structural elements of health as they relate to AI).
- Papers that look at trust in AI solutions
- Papers that look at the extent of the impact that population beliefs and public discourses/ rhetoric have on public perception of AI.
- Papers that look at determinants of trust in AI.
- Papers that look at risk and uncertainty as they relate to trust in AI

### A1.2 Exclusion criteria:

General

- Exclude review papers, pre-prints, books, book chapters, conference papers, thesis documents, editorials.
- Exclude papers not in English.
- Exclude papers with abstract only or full text only.
- Exclude policy briefs, commentaries.
- Exclude papers published prior to 2022.

Population

- Papers that look at trust from healthcare professionals, medical students, software developers/ engineers, ONLY.
- Ethical or philosophical papers that do not focus on public’s trust
- Theoretical papers exploring trust or developing trust frameworks

Context

- Exclude papers that ONLY look at contexts outside of healthcare (ie, AI in military; AI in art; AI in schools, etc), or that include healthcare and other contexts, but these are not differentiated in the text.

Focus

- Papers that focus on improving technology/ technology features to make AI more reliable, effective, accurate, etc
- Papers that focus on evaluating the reliability, effectiveness, accuracy and safety of AI
- Papers that look at trust in Robots that are not have AI technology.
- Papers that look at AI trustworthiness but not at public’s trust or public’s perceptions of AI
- Papers that look at expectation, perceptions etc but do not focus on the concepts of (Trust, Mistrust, Distrust, Confidence, Trustworthiness)
