# Supplementary material for: Trust, Risk, and Uncertainty Surrounding the Use of AI in Health Care: Scoping Review of Studies on Public Perception
Source: JMIR AI. 2026 Oct 30;5:e100747. doi: 10.2196/100747 (PMC13721850; doi:10.2196/100747)
Supplement: Supplementary file 3 [file ai-v5-e100747-s002.docx]

# Trust, Risk, and Uncertainty Surrounding the Use of Artificial Intelligence in Healthcare: A Scoping Review of Studies on Public Perception

## Appendix 2. Developed search strategies:

### A2.1 PubMed

(("Trust"[Title] OR "Mistrust"[Title] OR "Distrust"[Title] OR "Confidence"[Title] OR "Trustworthiness"[Title] OR "Trust"[MeSH Terms]) AND ("Artificial Intelligence"[Text Word] OR "Explainable AI"[Text Word] OR "AI-based"[Text Word] OR "AI-powered"[Text Word] OR "AI systems"[Text Word] OR "Human-AI"[Text Word] OR "AI-Human"[Text Word] OR "AI-assisted"[Text Word] OR "Fair-AI"[Text Word] OR "ai program*"[Text Word] OR "Medical AI"[Text Word] OR "ai algorithm*"[Text Word] OR "Machine learning"[Text Word] OR "neural network*"[Text Word] OR "deep learning"[Text Word] OR "natural language* processing"[Text Word] OR "rule based expert system*"[Text Word] OR "physical robot*"[Text Word] OR "Robotic process automation"[Text Word] OR "ChatGPT"[Text Word] OR "Gemini"[Text Word] OR "VertexAI"[Text Word] OR "Microsoft Copilot"[Text Word] OR "Artificial Intelligence"[MeSH Terms] OR "deep learning"[MeSH Terms] OR "Natural Language Processing"[MeSH Terms] OR "neural networks, computer"[MeSH Terms])) AND ((english[Filter]) AND (2022:2024[pdat]))

- Searched performed on 2024-10-26
- Retrieved 447 Results

### A2. 2 Scopus

( TITLE-ABS-KEY ( "Artificial Intelligence" OR "Explainable AI" OR "AI-based" OR "AI-powered" OR "AI systems" OR "Human-AI" OR "AI-Human" OR "AI-assisted" OR "Fair-AI" OR "ai program*" OR "Medical AI" OR "ai algorithm*" OR "Machine learning" OR "neural network*" OR "deep learning" OR "natural language* processing" OR "rule based expert system*" OR "physical robot*" OR "Robotic process automation" OR "ChatGPT" OR "Gemini" OR "VertexAI" OR "Microsoft Copilot" ) AND TITLE ( "Trust" OR "Mistrust" OR "Distrust" OR "Confidence" OR "Trustworthiness" ) AND TITLE-ABS-KEY ( "Health system*" OR healthcare OR "Healthcare system*" OR "Medical system*" OR "Public health" OR "Health Practice*" OR hospital* OR clinic* OR "Care home*" OR "Medical school*" OR "Health discipline*" OR telemedicine OR "Delivery of care" OR "Culturally competent care" OR "Health service*" OR "Digital Health" OR "Evidence-Based Medicine" OR "Culturally Competent Care" OR "Health Priorit*" OR "Health Resource*" OR "Population Health" OR "Quaternary Prevention" OR "Secondary Care" OR "Tertiary Healthcare" OR "Value-Based Health Care" OR "Attitude to Health" OR "Health Knowledge" OR "Health Attitude*" OR "Treatment Adherence" OR "Treatment Compliance" ) ) AND ( LIMIT-TO ( DOCTYPE , "ar" ) OR LIMIT-TO ( DOCTYPE , "cp" ) )

- Searched performed on 2024-10-26
- Retrieved 439 Results

### A2.3 Web of Science

("Artificial Intelligence" OR "Explainable AI" OR "AI-based" OR "AI-powered" OR "AI systems" OR "Human-AI" OR "AI-Human" OR "AI-assisted" OR "Fair-AI" OR "ai program*" OR "Medical AI" OR "ai algorithm*" OR "Machine learning" OR "neural network*" OR "deep learning" OR "natural language* processing" OR "rule based expert system*" OR "physical robot*" OR "Robotic process automation" OR "ChatGPT" OR "Gemini" OR "VertexAI" OR "Microsoft Copilot" (Title) or "Artificial Intelligence" OR "Explainable AI" OR "AI-based" OR "AI-powered" OR "AI systems" OR "Human-AI" OR "AI-Human" OR "AI-assisted" OR "Fair-AI" OR "ai program*" OR "Medical AI" OR "ai algorithm*" OR "Machine learning" OR "neural network*" OR "deep learning" OR "natural language* processing" OR "rule based expert system*" OR "physical robot*" OR "Robotic process automation" OR "ChatGPT" OR "Gemini" OR "VertexAI" OR "Microsoft Copilot" (Abstract) or "artificial intelligence" or "AI" (Topic)) AND ("Health system*" OR healthcare OR "Healthcare system*" OR "Medical system*" OR "Public health" OR "Health Practice*" OR hospital* OR clinic* OR "Care home*" OR "Medical school*" OR "Health discipline*" OR telemedicine OR "Delivery of care" OR "Culturally competent care" OR "Health service*" OR "Digital Health" OR "Evidence-Based Medicine" OR "Culturally Competent Care" OR "Health Priorit*" OR "Health Resource*" OR "Population Health" OR "Quaternary Prevention" OR "Secondary Care" OR "Tertiary Healthcare" OR "Value-Based Health Care" OR "Attitude to Health" OR "Health Knowledge" OR "Health Attitude*" OR "Treatment Adherence" OR "Treatment Compliance" (Title) or "Health system*" OR healthcare OR "Healthcare system*" OR "Medical system*" OR "Public health" OR "Health Practice*" OR hospital* OR clinic* OR "Care home*" OR "Medical school*" OR "Health discipline*" OR telemedicine OR "Delivery of care" OR "Culturally competent care" OR "Health service*" OR "Digital Health" OR "Evidence-Based Medicine" OR "Culturally Competent Care" OR "Health Priorit*" OR "Health Resource*" OR "Population Health" OR "Quaternary Prevention" OR "Secondary Care" OR "Tertiary Healthcare" OR "Value-Based Health Care" OR "Attitude to Health" OR "Health Knowledge" OR "Health Attitude*" OR "Treatment Adherence" OR "Treatment Compliance" (Abstract) or "Health system" OR healthcare OR "public health" (Topic)) AND (TI=("Trust" OR "Mistrust" OR "Distrust" OR "Confidence" OR "Trustworthiness")) AND (LIMIT: 2022-01-01 TO 2024-10-26)

- Searched performed on 2024-10-26
- Retrieved 210 Results

### A2. 4 PsycINFO

(((title: ("Health system*") OR title: (healthcare) OR title: ("Healthcare system*") OR title: ("Medical system*") OR title: ("Public health") OR title: ("Health Practice*") OR title: (hospital*) OR title: (clinic*) OR title: ("Care home*") OR title: ("Medical school*") OR title: ("Health discipline*") OR title: (telemedicine) OR title: ("Delivery of care") OR title: ("Culturally competent care") OR title: ("Health service*") OR title: ("Digital Health") OR title: ("Evidence-Based Medicine") OR title: ("Culturally Competent Care") OR title: ("Health Priorit*") OR title: ("Health Resource*") OR title: ("Population Health") OR title: ("Quaternary Prevention") OR title: ("Secondary Care") OR title: ("Tertiary Healthcare") OR title: ("Value-Based Health Care") OR title: ("Attitude to Health") OR title: ("Health Knowledge") OR title: ("Health Attitude*") OR title: ("Treatment Adherence") OR title: ("Treatment Compliance")) OR (abstract: ("Health system*") OR abstract: (healthcare) OR abstract: ("Healthcare system*") OR abstract: ("Medical system*") OR abstract: ("Public health") OR abstract: ("Health Practice*") OR abstract: (hospital*) OR abstract: (clinic*) OR abstract: ("Care home*") OR abstract: ("Medical school*") OR abstract: ("Health discipline*") OR abstract: (telemedicine) OR abstract: ("Delivery of care") OR abstract: ("Culturally competent care") OR abstract: ("Health service*") OR abstract: ("Digital Health") OR abstract: ("Evidence-Based Medicine") OR abstract: ("Culturally Competent Care") OR abstract: ("Health Priorit*") OR abstract: ("Health Resource*") OR abstract: ("Population Health") OR abstract: ("Quaternary Prevention") OR abstract: ("Secondary Care") OR abstract: ("Tertiary Healthcare") OR abstract: ("Value-Based Health Care") OR abstract: ("Attitude to Health") OR abstract: ("Health Knowledge") OR abstract: ("Health Attitude*") OR abstract: ("Treatment Adherence") OR abstract: ("Treatment Compliance")) OR (Index Terms: ("Health system*") OR Index Terms: (healthcare) OR Index Terms: ("Healthcare system*") OR Index Terms: ("Medical system*") OR Index Terms: ("Public health") OR Index Terms: ("Health Practice*") OR Index Terms: (hospital*) OR Index Terms: (clinic*) OR Index Terms: ("Care home*") OR Index Terms: ("Medical school*") OR Index Terms: ("Health discipline*") OR Index Terms: (telemedicine) OR Index Terms: ("Delivery of care") OR Index Terms: ("Culturally competent care") OR Index Terms: ("Health service*") OR Index Terms: ("Digital Health") OR Index Terms: ("Evidence-Based Medicine") OR Index Terms: ("Culturally Competent Care") OR Index Terms: ("Health Priorit*") OR Index Terms: ("Health Resource*") OR Index Terms: ("Population Health") OR Index Terms: ("Quaternary Prevention") OR Index Terms: ("Secondary Care") OR Index Terms: ("Tertiary Healthcare") OR Index Terms: ("Value-Based Health Care") OR Index Terms: ("Attitude to Health") OR Index Terms: ("Health Knowledge") OR Index Terms: ("Health Attitude*") OR Index Terms: ("Treatment Adherence") OR Index Terms: ("Treatment Compliance"))) AND ((Year: [2022 TO 2024] OR TestYear: [2022 TO 2024]))) AND (((title: ("Trust" OR "Mistrust" OR "Distrust" OR "Confidence" OR "Trustworthiness")) OR (abstract: ("Trust" OR "Mistrust" OR "Distrust" OR "Confidence" OR "Trustworthiness")) OR (Index Terms: ("Trust" OR "Mistrust" OR "Distrust" OR "Confidence" OR "Trustworthiness"))) AND ((Year: [2022 TO 2024] OR TestYear: [2022 TO 2024]))) AND (((title: ("Artificial Intelligence") OR title: ("Explainable AI") OR title: ("AI-based") OR title: ("AI-powered") OR title: ("AI systems") OR title: ("Human-AI") OR title: ("AI-Human") OR title: ("AI-assisted") OR title: ("Fair-AI") OR title: ("ai program*") OR title: ("Medical AI") OR title: ("ai algorithm*") OR title: ("Machine learning") OR title: ("neural network*") OR title: ("deep learning") OR title: ("natural language* processing") OR title: ("rule based expert system*") OR title: ("physical robot*") OR title: ("Robotic process automation") OR title: ("ChatGPT") OR title: ("Gemini") OR title: ("VertexAI") OR title: ("Microsoft Copilot")) OR (abstract: ("Artificial Intelligence") OR abstract: ("Explainable AI") OR abstract: ("AI-based") OR abstract: ("AI-powered") OR abstract: ("AI systems") OR abstract: ("Human-AI") OR abstract: ("AI-Human") OR abstract: ("AI-assisted") OR abstract: ("Fair-AI") OR abstract: ("ai program*") OR abstract: ("Medical AI") OR abstract: ("ai algorithm*") OR abstract: ("Machine learning") OR abstract: ("neural network*") OR abstract: ("deep learning") OR abstract: ("natural language* processing") OR abstract: ("rule based expert system*") OR abstract: ("physical robot*") OR abstract: ("Robotic process automation") OR abstract: ("ChatGPT") OR abstract: ("Gemini") OR abstract: ("VertexAI") OR abstract: ("Microsoft Copilot")) OR (Index Terms: ("Artificial Intelligence") OR Index Terms: ("Explainable AI") OR Index Terms: ("AI-based") OR Index Terms: ("AI-powered") OR Index Terms: ("AI systems") OR Index Terms: ("Human-AI") OR Index Terms: ("AI-Human") OR Index Terms: ("AI-assisted") OR Index Terms: ("Fair-AI") OR Index Terms: ("ai program*") OR Index Terms: ("Medical AI") OR Index Terms: ("ai algorithm*") OR Index Terms: ("Machine learning") OR Index Terms: ("neural network*") OR Index Terms: ("deep learning") OR Index Terms: ("natural language* processing") OR Index Terms: ("rule based expert system*") OR Index Terms: ("physical robot*") OR Index Terms: ("Robotic process automation") OR Index Terms: ("ChatGPT") OR Index Terms: ("Gemini") OR Index Terms: ("VertexAI") OR Index Terms: ("Microsoft Copilot"))) AND ((Year: [2022 TO 2024] OR TestYear: [2022 TO 2024])))

- Searched performed on 2024-10-26

Retrieved 192 Results
