# Supplementary material for: Trust, Risk, and Uncertainty Surrounding the Use of AI in Health Care: Scoping Review of Studies on Public Perception
Source: JMIR AI. 2026 Oct 30;5:e100747. doi: 10.2196/100747 (PMC13721850; doi:10.2196/100747)
Supplement: Supplementary file 4 [file ai-v5-e100747-s006.docx]

# Trust, Risk, and Uncertainty Surrounding the Use of Artificial Intelligence in Healthcare: a Scoping Review of Studies on Public Perception

## Appendix 6. Table S4.

Table S4. Trust definitions based on identified themes

| Definition | Wording/ Language | Trusted Entity/Tool in Question |
| --- | --- | --- |
| *Trust in technology (n = 8)* | | |
| Defines Trust in ChatGPT as a user’s willingness to take chances based on the recommendations made by this technology (1). | Implies that there is a luck factor which may mean that there is uncertainty and thus risk involved. | ChatGPT for healthcare-related purposes. |
| Trust is the confidence users have in ChatGPT’s reliability, accuracy, and ethical standards when providing health-related information (2). | Emphasis is on technology and its performance. | ChatGPT ability to provide accurate, useful, and ethical health information. |
| Trust is defined as the degree to which an individual perceives that smart healthcare services are dependable, reliable, and trustworthy in supporting one’s healthcare activities (3). | Emphasis on technology and its performance.  Emphasizes that trust is based on individual and subjective experience. | ChatGPT |
| Trust evaluation encompasses not only the system’s ability but also its perceived benevolence and integrity, thus encompassing the societal dimensions of AI systems. This framework evaluates trust based on three factors – the system’s ability, benevolence, and integrity (4). | It goes beyond the system’s ability and performance and may imply that the system may demonstrate integrity and benevolence. | AI in Health care system |
| We define trust as an individual’s belief in a chatbot agent to help them achieve specific goals (5). | Emphasis on technology and its performance. | AI Chatbot |
| The beliefs that a specific technology has the attributes necessary to perform as expected in a given situation in which negative consequences are possible” (6). | Emphasis the technology and its performance. As well it recognizes risk (ie, negative consequences are possible). | AI-based Chatbots for Self  Diagnosis (AICSD) |
| Trust in technology is the belief that a tool, machine, or equipment will not fail (7). | Emphasis is on technology and its performance. | Mental Health Apps powered by AI |
| *Concepts adjacent to trust (n = 3)* | | |
| User acceptance is defined as the intention to use technology and is considered a direct antecedent of actual user behavior (8). | May imply that some level of trust already exists.. The motivations to use technology are unknown from acceptance alone. | AI chatbots and AI avatars |
| Trustworthiness is a characteristic of an agent that denotes the agent’s competence to perform an action and her moral attitude or commitment towards those who depend on her to perform said action (9). | Acknowledgement that the onus is not on the trustee. Emphasis on performance but also on the moral attitude of the trustor towards the trustee. | AI-based cardiotocography (CTG) in the context of intrapartum care |
| Medical mistrust, ie, a tendency to distrust healthcare providers, institutions, or the system (10). | Focus on the healthcare system and not AI systems (Mistrust in the medical system may lead to trust AI over medical professionals). The use of the word tendency may suggest a general natural inclination which could imply that context factors or characteristics of entities involved are not as important. | Health system |
| *Specialized trust (n = 3)* | | |
| *Experiential trust:* Trust in new technology is learned through familiarity and experience (11).  *Dispositional trust:* The willingness to trust can be construed as a disposition or an individual propensity (11).  *Situational trust:* Trust is malleable, allowing for conceptualization and measurement as a state. To understand the variability of trust in FRT, researchers have encouraged a careful examination of risks and benefits in specific contexts or scenarios. Public spaces, schools, and hospitals were identified as specific contexts (11). | All of these forms of specialized trust help understand different mechanisms for trust formation and deterioration. All of these trust forms may occur simultaneously or not, but it is not clear as to how trust formation may (or not) be compounded. Emotional trust is particularly interesting considering that it may speak to the role of anthropomorphic characteristics in shaping the public’s trust in AI.. | Facial recognition technology (the AI tool) and AI-powered surveillance, relevant local authorities (ie, people, institutions, organizations, and health providers). |
| *Relational trust* is conceptualized in this study as an individual’s willingness and confidence to rely on a healthcare organization that provides AI chatbot services (12). |  | Chatbot |
| *Cognitive trust* (in competence), which refers to a patient’s rational expectation that the doctor possesses the necessary expertise to provide effective recommendations (13).  Emotional trust, characterized by a patient’s sense of security, comfort, and satisfaction with the recommendations provided by the doctor (13). |  | ChatGPT AI doctor |
| *Trust in general (n = 6)* | | |
| Trust is defined according to the ability to determine who to trust, and represents the willingness of a party to be vulnerable to the actions of another party based on the expectation that the other will perform a particular action important to the trustor, irrespective of the ability to monitor or control that other party (6,14,15). | Emphasis on vulnerability as needed to trust. Emphasis on the trustor being benevolent.  Acknowledgement that one party may have no control over the other, which could mean that at times there are levels of uncertainty and risk within the trusting relationship. AI can be monitored or controlled, which relates to governance and oversight. | AI-based COVID-tracing app, AICSD, Trust in AI-based medical consultation platforms. |
| The willingness to make oneself vulnerable to another based on a judgment of similarity of intentions or values. Trust is important in helping to facilitate choices in situations characterized by uncertainty, vulnerability, and perceived risk, where the “motives, intentions, and prospective actions of others” are unknown (16). | Emphasis on vulnerability as needed to trust. Emphasis on the trustor being benevolent.  Acknowledges the role of risk and uncertainty in trust. | AI enabled autonomous technologies: robotic surgery |
| Trust is defined as the act of accepting vulnerability, in which the doctor is expected to act in the patient’s best interests and provide care (8). | Emphasis on vulnerability as needed to trust. Emphasis on the trustor being benevolent. | AI chatbots and AI avatars |
| Trust is a psychological state broadly defined based on 3 main dimensions, namely, benevolence, integrity, and ability (17). | Acknowledges that trust is psychological which could relate to the idea that behaviors are shaped by thinking mechanisms. | AI product |

1.	Choudhury A, Shamszare H. Investigating the Impact of User Trust on the Adoption and Use of ChatGPT: Survey Analysis. J Med Internet Res. 2023 Jun 14;25:e47184. doi:10.2196/47184

2.	Alanezi F. Factors influencing patients’ engagement with ChatGPT for accessing health-related information. Critical Public Health. 2024 Dec 31;34(1):1–20. doi:10.1080/09581596.2024.2348164

3.	Liu K, Tao D. The roles of trust, personalization, loss of privacy, and anthropomorphism in public acceptance of smart healthcare services. Computers in Human Behavior. 2022 Feb;127:107026. doi:10.1016/j.chb.2021.107026

4.	Novozhilova E, Mays K, Paik S, Katz JE. More Capable, Less Benevolent: Trust Perceptions of AI Systems across Societal Contexts. Machine Learning and Knowledge Extraction. 2024 Mar;6(1):1. doi:10.3390/make6010017

5.	Park G, Chung J, Lee S. Human vs. machine-like representation in chatbot mental health counseling: the serial mediation of psychological distance and trust on compliance intention. Curr Psychol. 2024 Feb 1;43(5):4352–63. doi:10.1007/s12144-023-04653-7

6.	Prakash AV, Das S. (Why) Do We Trust AI?: A Case of AI-based Health Chatbots. Australasian Journal of Information Systems. 2024 May 15;28. doi:10.3127/ajis.v28.4235

7.	Shan Y, Ji M, Xie W, Lam KY, Chow CY. Public Trust in Artificial Intelligence Applications in Mental Health Care: Topic Modeling Analysis. JMIR Hum Factors. 2022 Dec 2;9(4):e38799. doi:10.2196/38799

8.	Mayer CJ, Mahal J, Geisel D, Geiger EJ, Staatz E, Zappel M, et al. User preferences and trust in hypothetical analog, digitalized and AI-based medical consultation scenarios: An online discrete choice survey. Computers in Human Behavior. 2024 Dec;161:108419. doi:10.1016/j.chb.2024.108419

9.	Dlugatch R, Georgieva A, Kerasidou A. Trustworthy artificial intelligence and ethical design: public perceptions of trustworthiness of an AI-based decision-support tool in the context of intrapartum care. BMC Med Ethics. 2023 Jun 20;24(1):42. doi:10.1186/s12910-023-00917-w

10.	Gaube S, Biebl I, Engelmann MKM, Kleine AK, Lermer E. Comparing preferences for skin cancer screening: AI-enabled app vs dermatologist. Social Science & Medicine. 2024 May;349:116871. doi:10.1016/j.socscimed.2024.116871

11.	Choung H, David P, Ling TW. Acceptance of AI-powered facial recognition technology in surveillance scenarios: Role of trust, security, and privacy perceptions. Technology in Society. 2024 Dec 1;79:102721. doi:10.1016/j.techsoc.2024.102721

12.	Huang M, and Ki EJ. Examining the Effect of Anthropomorphic Design Cues on Healthcare Chatbots Acceptance and Organization-Public Relationships: Trust in a Warm Human Vs. a Competent Machine. International Journal of Human–Computer Interaction. 2024 Dec 16;40(24):8781–93. doi:10.1080/10447318.2023.2290378

13.	Qin H, Zhu Y, Jiang Y, Luo S, Huang C. Examining the impact of personalization and carefulness in AI-generated health advice: Trust, adoption, and insights in online healthcare consultations experiments. Technology in Society. 2024 Dec;79:102726. doi:10.1016/j.techsoc.2024.102726

14.	Luo Y, Li X, Ye Q. The Impact of Privacy Calculus and Trust on User Information Participation Behavior in AI-based Medical Consultation - The Moderating Role of Gender. Journal of Electronic Commerce Research, 24 [Internet]. 2023. Available from: http://ojs.jecr.org/jecr/sites/default/files/2023vol24no1_Paper4.pdf

15.	Sousa S, Kalju T. Modeling Trust in COVID-19 Contact-Tracing Apps Using the Human-Computer Trust Scale: Online Survey Study. JMIR Hum Factors. 2022 Jun 13;9(2):e33951. doi:10.2196/33951 PubMed PMID: 35699973; PubMed Central PMCID: PMC9202513.

16.	Horowitz MC, Kahn L, Macdonald J, Schneider J. Adopting AI: how familiarity breeds both trust and contempt. AI & Soc. 2024 Aug;39(4):1721–35. doi:10.1007/s00146-023-01666-5

17.	Rieger T, Kugler L, Manzey D, Roesler E. The (Im)perfect Automation Schema: Who Is Trusted More, Automated or Human Decision Support? Hum Factors. 2024 Aug;66(8):1995–2007. doi:10.1177/00187208231197347
